# Supplementary material for: Healthcare consumers’ perceptions of incentive-linked prescribing: A scoping review
Source: PLOS Glob Public Health. 2024 Jun 27;4(6):e0003026. doi: 10.1371/journal.pgph.0003026 (PMC11210849; doi:10.1371/journal.pgph.0003026)
Supplement: S1 File — (DOCX) [file pgph.0003026.s001.docx]

**Literature search strategy**

**Scopus (n=445)**

(TITLE-ABS-KEY(physician OR physicians OR physician* OR doctor OR doctors) AND TITLE-ABS(patient OR patients) AND TITLE-ABS-KEY(“pharmaceutical industry” OR "drug company" OR "drug companies" OR "drug manufactur*" OR “drug industry”) AND TITLE-ABS(financial OR graft OR influence* OR freebie* OR gift OR gifts OR monetary OR cash OR money)) AND PUBYEAR > 2002 AND PUBYEAR < 2024 AND ( LIMIT-TO ( DOCTYPE,"ar" ) OR LIMIT-TO ( DOCTYPE,"re" ) OR LIMIT-TO ( DOCTYPE,"ed" ) ) AND ( LIMIT-TO ( LANGUAGE,"English" ) )

**Medline (n=77)**

Ovid MEDLINE(R) and Epub Ahead of Print, In-Process, In-Data-Review & Other Non-Indexed Citations and Daily <1946 to September 11, 2023>

1 Gift Giving/ 1708

2 Drug Industry/ 35019

3 1 and 2 522

4 patient*.ti,ab,kf. 8188156

5 Patients/ 24328

6 public.ti,ab. 593506

7 4 or 5 or 6 8650625

8 1 and 2 and 7 112

9 (comment or editorial or news or newspaper article).pt. 1696172

10 (letter not (letter and randomized controlled trial)).pt. 1222049

11 9 or 10 2403074

12 8 not 11 97

13 limit 12 to (english language and yr="2002 -Current") 77

**Embase (42)**

Embase <1974 to 2023 Week 36>

1 Gift Giving/ 1253

2 Drug Industry/ 92160

3 1 and 2 360

4 patient*.ti,ab,kf. 11972863

5 Patients/ 1057052

6 public.ti,ab. 708395

7 4 or 5 or 6 12493306

8 1 and 2 and 7 61

9 limit 8 to (article or article in press or "review") 53

10 limit 9 to (english language and yr="2002 -Current") 42
