# Supplementary material for: Healthcare consumers’ perceptions of incentive-linked prescribing: A scoping review
Source: PLOS Glob Public Health. 2024 Jun 27;4(6):e0003026. doi: 10.1371/journal.pgph.0003026 (PMC11210849; doi:10.1371/journal.pgph.0003026)
Supplement: S2 File — (DOCX) [file pgph.0003026.s002.docx]

| **S2 File: Literature Matrix** | | | | | | | | | | | | |  |
| --- | --- | --- | --- | --- | --- | --- | --- | --- | --- | --- | --- | --- | --- |
| **Themes** | Ammous et al (2017) | Crigger et al (2009) | Gillani et al (2022) | Goff et al (2008) | Grande et al (2012) | Green et al (2012) | Hwong, Sah & Lehmann (2017) | Jastifer & Roberts (2009) | Kaur et al (2022) | Semin et al (2006) | Tattersal,Dimoskka & Gan (2009) | Wise & Rodseth (2013) | Perry et al. (2014) |
| Higher trust in physicians who receive no payments from the pharma industry |  |  |  |  |  |  | X |  |  |  |  |  |  |
| Physicians who have a financial relationship with the pharma industry do not work in the best interest of patients |  |  |  |  |  |  | X |  |  |  |  |  |  |
| Low trust/even public disclosure of pharma payments decreases patients' trust, although important | X |  | X |  |  |  | X |  | X | X | X | X |  |
| Patients have a good awareness of pharmaceutical incentivisation for doctors | X | X | X |  | X |  |  |  |  | X |  |  |  |
| Suspicion about doctor-PSR interaction | X |  |  |  |  |  |  |  | X | X |  |  |  |
| Awareness about incentive-types | X | X | X |  |  |  |  |  | X |  |  |  |  |
| Incentivisation affects prescribing behaviour | X | X | X |  |  | X |  | X | X | X | X | X |  |
| Pharmaceutical incentivisation is unethical | X | X | X |  |  | X |  |  | X | X |  | X |  |
| Doctor-PSR interaction makes patients wait longer | X |  | X |  |  | X |  |  |  |  |  |  |  |
| Need for educating the public about ILP | X |  |  |  |  |  |  |  | X |  |  |  |  |
| Need for stronger health-system regulation | X |  |  |  |  |  |  |  | X | X |  |  |  |
| Doctors' sensitisation for self-regulation for patients' trust deficit | X |  |  |  |  |  |  |  |  |  |  | X |  |
| Patients want transparency in the doctor-pharma relationship |  |  |  | X |  |  |  |  | X |  | X |  |  |
| Lack of awareness about ILP |  |  |  |  |  |  |  |  | X |  | X |  | X |
| The patient believed that prescribing practices influenced by PSRs |  | X | X | X |  |  |  |  | X | X |  |  |  |
| Educational and Low cost gifts can ethically be taken |  | X |  |  |  |  |  |  | X | X |  |  |  |
| It is beleived that cost is effected by Gifts |  | X |  |  |  |  |  |  | X | X |  |  |  |
| Influence of PRs for unnecessry prescription of Antibiotics |  |  | X |  |  |  |  |  |  |  |  |  |  |
| Trust on doctors [communication, knowledge, medication and experience] |  |  |  | X |  |  |  |  |  |  |  |  |  |
| Docrtors must share options of medicine, cost, pros and cons of medicines |  |  |  | X |  |  |  |  |  |  |  |  |  |
| Low awareness of the gift-related practices of physicians |  |  |  |  |  | X |  | X |  |  |  |  | X |
| Acceptance of gifts will lower the trust |  |  |  |  | X | X |  |  |  |  |  |  |  |
| Gifts of larger value lowers the trust of patients in their HCP |  |  |  |  |  | X |  |  |  |  |  |  |  |
| Acceptance of gifts signifies low trust in physicians and the healthcare system |  |  |  |  | X |  |  |  |  |  |  |  | X |
| Patients think gifts of nominal value are acceptable |  |  |  |  |  |  |  | X |  |  |  |  |  |
| People with higher education levels have awareness of the gifts |  |  |  |  |  |  |  | X |  |  |  |  |  |
