# Supplementary material for: Healthcare consumers’ perceptions of incentive-linked prescribing: A scoping review
Source: PLOS Glob Public Health. 2024 Jun 27;4(6):e0003026. doi: 10.1371/journal.pgph.0003026 (PMC11210849; doi:10.1371/journal.pgph.0003026)
Supplement: Attachment — Submitted filename: Response to Reviewers.docx [file pgph.0003026.s004.docx]

**Response to reviewers**

**Editor**

Please address the concerns raised by the reviewer, specifically:
**Comment: state the design of the reviewed studies**

Response: the design of the reviewed studies has been described on page 9, lines 156 -160.
**Comment: in discussion, expand on both ethical and unethical promotional activities as well as perceived incentives within the framework of ethical and unethical practices (as defined by Federal anti-kickback laws).**

Response: Using the Federal ani-kickback law and the American Medical Association’s code of ethics, we have explained patients’ perceptions of acceptable/unacceptable incentives. Please see pages 18-19, lines 269 – 282 (manuscript with track changes).

**Reviewer #1**

**Comment: The title and the objective highlighted in this scoping review is important to understand pharmaceutical promotion from the patient and general public perspective. However, I recommend some modifications in order to enrich the work. First, use a uniform referencing style or put the reference citation before the full stop. Second, reduce the unnecessary use of pronouns and present perfect tenses.**

Response: Thank you for highlighting this issue. We performed a thorough proofreading of the manuscript to fix the editorial and grammatical errors, you highlighted.

Furthermore, address the point listed below.
Introduction
**Comment #1: the idea stated from line 56 to 61 is too basic for a research article.**

Response: We agree with your suggestion. We have now removed lines 56-61 from the manuscript.

Methods
**Comment #2: why did you only include articles publish from 2003 onwards?**

Response: You have indeed raised a very important question. We deemed studies before 2000 as outdated because codes of ethics and policies are dynamic. Therefore, looking at what has been published in the last 20 years about patient perceptions of pharmaceutical incentivisation, to us, was more appropriate.

**Comment #3: If possible, remove box 2 and attach it as additional files.**

Response: Thanks for the suggestion. We have removed box 2 and provided our search strategy as a supplemental file.

**Comment #4: why didn’t you scrutinize articles by study design in stage 4?**

Response: Thank you for asking this important question. We indeed scrutinized articles by study design at stage 4, but unfortunately somehow did not mention it in the manuscript. We have now inserted this information on page 10, lines 157-161 (manuscript with track changes).

Discussion
**#5: Discuss the ethical promotional vs unethical promotional activities. Otherwise, some of the activities highlighted by this study could not be considered as incentivizing the physicians. For example, Providing product sample is ethical promotional activity and as long as the physician did not use the sample for treatment purpose is not an incentive.**

Response: Thank you for the suggestion. Using the Federal ani-kickback law and the American Medical Association’s code of ethics, we have explained patients’ perceptions of acceptable/unacceptable incentives, which clarifies that drug samples/notepads/pens may not be considered incentives. Please see pages 18-19, lines 269 – 282 (manuscript with track changes).

**Reviewer #2**

**The authors have done justice to this scoping review which interogatted the knowledge and views of healthcare consumers (patients) about the interaction between physicians and the pharmarceutical industry.
The manuscript followed the steps recommended for a scoping review and the deductions were spot on.**

Response: We are grateful for reviewing our manuscript and providing us with highly positive feedback.
